# RNA expression patterns in serum microvesicles from patients with glioblastoma multiforme and controls

**DOI:** 10.1186/1471-2407-12-22

**Published:** 2012-01-17

**Authors:** Mikkel Noerholm, Leonora Balaj, Tobias Limperg, Afshin Salehi, Lin Dan Zhu, Fred H Hochberg, Xandra O Breakefield, Bob S Carter, Johan Skog

**Affiliations:** 1 Department of Neurology, Neurosurgery and Radiology, Massachusetts General Hospital and Program in Neuroscience, Harvard Medical School, Boston, MA 02114, USA; 2 Exosome Diagnostics, Inc., Lasker Biomedical Research Building, Columbia University Medical Center, 3960 Broadway, Suite 540, New York, NY 10032, USA; 3 Department of Neuroscience and Pharmacology, Rudolf Magnus Institute of Neuroscience, University Medical Center Utrecht, Utrecht, The Netherlands; 4 Division of Neurological Surgery, UCSD School of Medicine, San Diego, CA 92103, USA

**Keywords:** Exosomes, Microvesicles, Microarray, Biomarkers, Serum, Glioma

## Abstract

**Background:**

RNA from exosomes and other microvesicles contain transcripts of tumour origin. In this study we sought to identify biomarkers of glioblastoma multiforme in microvesicle RNA from serum of affected patients.

**Methods:**

Microvesicle RNA from serum from patients with de-novo primary glioblastoma multiforme (N = 9) and normal controls (N = 7) were analyzed by microarray analysis. Samples were collected according to protocols approved by the Institutional Review Board. Differential expressions were validated by qRT-PCR in a separate set of samples (N = 10 in both groups).

**Results:**

Expression profiles of microvesicle RNA correctly separated individuals in two groups by unsupervised clustering. The most significant differences pertained to down-regulated genes (121 genes > 2-fold down) in the glioblastoma multiforme patient microvesicle RNA, validated by qRT-PCR on several genes. Overall, yields of microvesicle RNA from patients was higher than from normal controls, but the additional RNA was primarily of size < 500 nt. Gene ontology of the down-regulated genes indicated these are coding for ribosomal proteins and genes related to ribosome production.

**Conclusions:**

Serum microvesicle RNA from patients with glioblastoma multiforme has significantly down-regulated levels of RNAs coding for ribosome production, compared to normal healthy controls, but a large overabundance of RNA of unknown origin with size < 500 nt.

## Background

Cancer molecular diagnostics is becoming increasingly important with the accumulating knowledge of the molecular mechanisms underlying various types of cancers and the implications for treatment option selection and prognosis. For patients with glioblastoma multiforme (GBM), treatment planning currently takes into account radiographic imaging, which documents volume and location of disease [1], and in some cases mutational analysis [2], methylation status of genomic DNA with particular emphasis on the DNA repair gene for methyl guanidine methyl transferase (MGMT [3]) and gene expression patterns of the tumour, which allows the broad categorization of tumours that are histologically similar into molecular subtypes [4]. To date most molecular studies have utilized primary explant cultures or frozen, formalin fixed tumour tissue derived at the time of surgical resection. These methods have the disadvantage that the part of the tumour specimen chosen for analysis may not represent the rest of the tumour, and the molecular profile of the recurrent tumour may be very different from the original biopsy. It would be very useful to have a way to monitor and evaluate the tumour gene expression pattern over time in a non-invasive assay, such as through a blood sample test. Over the past few years a growing list of studies has reported on the ability to use expression profiling tests on RNA derived from blood samples to differentiate between healthy controls and patients with certain types of cancer [5-8], to classify separate patient populations [9] or to predict clinical outcome [10]. The ability to conduct nucleic acid expression profiling assays on a blood sample rather than on tumours has a wide range of implications for patient welfare, including the ability to conduct longitudinal disease monitoring in situations where tumour tissue is not easily accessible or one is trying to sample metastatic cancer. Because the blood harbors nucleic acid of both tumour and non-tumour origin, it is possible that this approach may capture not only direct nucleic acid changes seen in the tumour cells, but also a component of the host response to the presence of tumour. For example, studies so far have reported on RNA extracted from Peripheral Blood Mononuclear Cells (PBMC) or other fractions of circulating blood cells where changes in the cell RNA profile appears to represent the host's response to the malignancy [5] rather than the tumour itself. Different groups have isolated RNA from circulating tumour cells [11] and from cell-free body fluids [12]. Given the short half-life of unprotected RNA in serum [13], it is likely that most of the cell-free RNA is protected in the exosomes/microvesicle fraction or in the case of microRNAs (miRNAs) by protein complexes in the blood [14,15].

Microvesicles are very stable and can protect cell-free RNA stored in the freezer for many years. This is a great advantage compared to analyzing circulating tumour cells where the blood needs to be processed within hours of collection. In addition, circulating tumour cells have not yet been described in glioma patients [16]. In this study, full microarray analysis was carried out on mRNA isolated from serum microvesicles (including exosomes and other shedding microvesicles [17]) from GBM patients and controls to test the hypothesis that this mRNA could be used to reflect tumour-associated changes in the exosomal/microvesicle fraction of serum RNA. RNA species showing differential expression were chosen for quantitative reverse transcriptase (qRT-PCR) validation. This study is the first to report the ability to differentiate GBM patients from normal controls based on a gene expression blood test and the first to report differential expression analysis using RNA extracted from exosomes/microvesicles isolated from clinical patient serum samples, as compared to controls.

## Methods

### Clinical samples

Blood samples from patients diagnosed with *de-novo *primary GBM were collected immediately prior to surgery (before opening of the *dura mater) *into a BD Vacutainer SST (#367985) at Massachusetts General Hospital (MGH). Patients were following standard of care at MGH, including fasting prior to surgery and most of the patients were treated with steroids to alleviate vasogenic edema and pain. Blood from normal healthy controls was collected from de-identified volunteers recruited at the MGH blood bank. All samples were collected with informed consent according to the appropriate protocols approved by the Institutional Review Board at MGH. The blood was left to clot for 30 min at room temperature (r.t.) and serum was isolated, according to manufacturer's recommendations within 2 h of collection. Serum was filtered by slowly passing it through a 0.8 μm syringe filter (Millopore, Billerica, MA, USA) and aliquoted into 1.8 mL cryotubes (Fisher Scientific, Waltham, MA, USA) and stored at -80°C until use.

### Isolation of microvesicle RNA

Isolation of RNA from microvesicles was performed as previously described [18] with a few modifications. Briefly, 1 mL of serum was transferred to an ultracentrifuge tube, diluted 1:3 with cold PBS and centrifuged at 120,000 g for 80 min at 8°C and the supernatant was carefully aspirated off without disturbing the microvesicle pellet. The pellet was resuspended, treated for 15 min with 4 U of DNase I (Ambion, Austin, TX, USA) (in 25 μL of the accompanying buffer), 700 μL miRNeasy lysis buffer (Qiazol Reagent) (Qiagen, Valencia, CA, USA) was then added to the tube and the RNA was isolated following the manufacturer's recommendations. After elution of the RNA from the column in 30 μL nuclease-free water (Ambion), the RNA was precipitated by adding 2.5 volumes 100% EtOH, 1/10 3 M sodium acetate (pH 5.2) and incubated at -20°C for 1 h. Samples were then centrifuged for 20 min at 16,000 g and the supernatant was removed. The pellet was left to dry at r.t. and dissolved in 14 μL nuclease-free water and stored at -80°C until needed. RNA quality and concentration was assessed with the Agilent Bioanalyzer RNA Pico Chip and the Nanodrop 2000 (Thermo Scientific, Wilmington, DE, USA).

### Linear amplification and array hybridization

Linear amplification and hybridization to Agilent microarrays was carried out by Miltenyi, according to manufacturer's recommendations. Briefly, exoRNA was linearly amplified and fluorescently labeled with Cy3 using Low Input Quick Amp Labeling Kit (Agilent, Santa Clara, CA, USA) and 1.4 μg amplified RNA was hybridized to Agilent 4×44K Human Microarrays, washed and scanned. Raw data was generated by image analysis using Feature Extraction (Agilent).

### Microarray data analysis

The raw data exported from Agilent Feature Extraction v9.1 was pre-processed and normalized using R/Bioconductor and the packages *limma*, *Agi4×44PreProcess *and *vsn *(see R script in the Additional file 1 information for details). Where not mentioned in the text, the data from quartile normalization after background subtraction were used.

To reduce the risk that the normalization procedure introduced unintended biases or artifacts, we normalized the data in three different ways using: 1) variance stabilized normalization (VSN); 2) quartile normalization with background subtraction; and 3) quartile normalization without background subtraction. Although there were small differences between the three methods many of the same genes turned out to be dysregulated regardless of the applied normalization.

Clustering analysis, heat maps and Linear Discriminant Analysis of the normalized data was done using dChip (http://biosun1.harvard.edu/complab/dchip). Normalized data was transferred to Excel and filtered with various criteria, as described in the text. Gene lists of interest were uploaded and analyzed with the online Gene Ontology Tool DAVID 6.7 http://david.abcc.ncifcrf.gov/. The raw data and the quartile normalized mean data with background subtraction has been deposited in GEO with accession# GSE24084.

### Reverse transcription and qPCR analysis

Twelve μL of the RNA isolated from 1 mL of serum were reverse transcribed using Superscript VILO cDNA synthesis kit (Invitrogen, Carlsbad, CA, USA), according to manufacturer's recommendations. Samples were then preamplified using the TaqMan^® ^PreAmp Master Mix (Applied Biosystems, Carlsbad, CA, USA). Briefly, 12.5 μL of the cDNA was added to the PreAmp Master Mix together with all the genes of interest and pre- amplified for 14 cycles, according to the manufacturer's recommendations. The samples were then diluted 1:10 and TaqMan qRT-PCR was performed on all samples for all the selected genes. The amplification was performed using ABI PRISM 7500 with the following program: 50°C, 2 min; 95°C, 10 min; 40 cycles of 95°C, 15 s, 60°C, 1 min on standard mode. Additional file 2: Table S1 contains a list of all the commercially available and custom made probes used.

## Results

### Unsupervised clustering separates GBM patients from controls in expression array analysis

**Figure 1:**
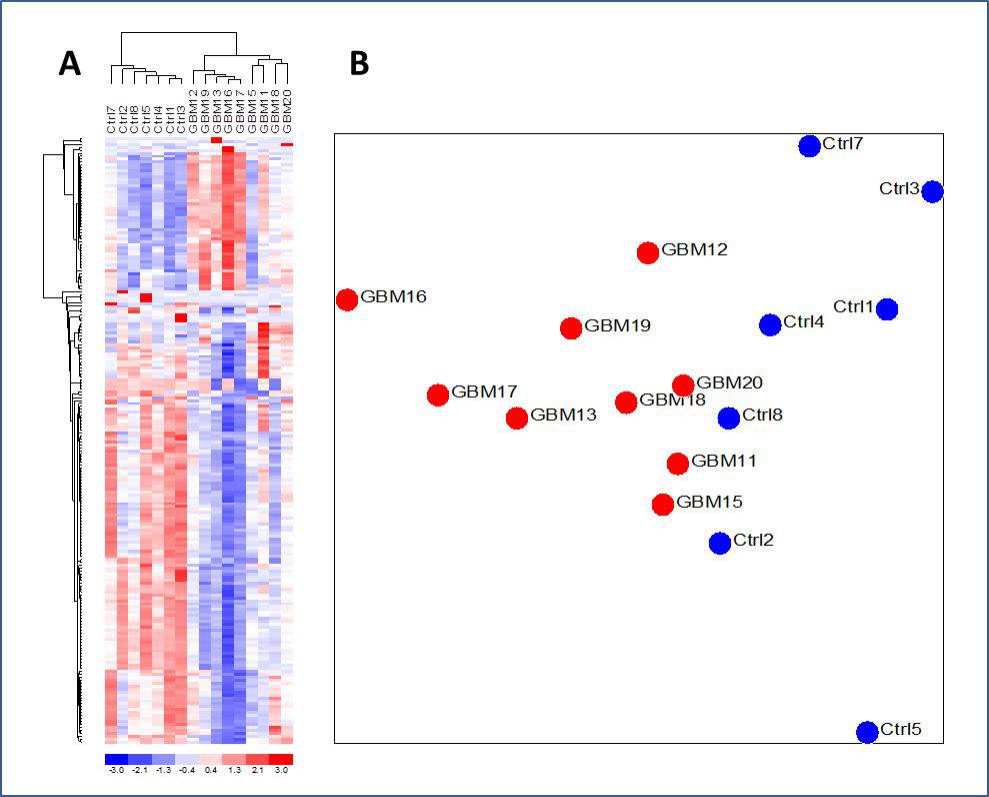
**A total of 206 genes were selected without application of *t*-test by filtering for high signal intensity (> 6 in 30% of samples) and high variation between samples (stdev > 0.8 across all 16 samples)**. A) A heat map and dendrogram showing perfect unsupervised clustering of the samples based on these 206 genes. B) Principal Component Analysis (PCA) of the same 206 genes as in A.

Exosomes and other microvesicles (less than 0.8 μm in diameter, see methods) were isolated from serum samples from 9 GBM patients immediately prior to tumour removal and from 7 normal healthy controls. RNA from this microvesicle fraction (exoRNA) was extracted, amplified by linear amplification, labeled and hybridized to Agilent microarrays containing 44,000 (44 K) capture probes against essentially all genes in the human genome. The raw data was background corrected and normalized between samples, as described in the Methods section. To investigate whether the two sets of samples could be separated by unsupervised clustering (without prior knowledge of the identity of each sample), we filtered the data to only include probes for which at least 4 out of 16 samples had a high signal intensity (intensity > 6) and for which the variation across all samples was high (Std. dev. > 0.8). This approach effectively excluded probes that displayed constant intensity in all samples and therefore did not contribute to distinguishing between the two groups. The selected subset of 206 probes thus displayed both variation and intensity, which are prerequisites for contributing to discrimination between the two groups. When the signal from these probes was analyzed with unsupervised clustering it perfectly separated the GBMs from the controls as illustrated by the heat map and dendrogram in Figure 1A. The sample dendrogram at the top of the heat map has two primary branches illustrating the perfect separation the GBM samples from the Normal Controls. The gene dendrogram to the left of the heat map separates the genes that are up-regulated from those that are down-regulated in GBM samples, respectively. The observation that the data clustered perfectly into two distinct groups without any pre-selection of genes based on *t*-test analysis indicates that there are significant differences in the expression profiles of the GBM exoRNA and the normal control exoRNA, even though the sample set in the current study is too small to pass formal power calculation criteria [19].

**Figure 2:**
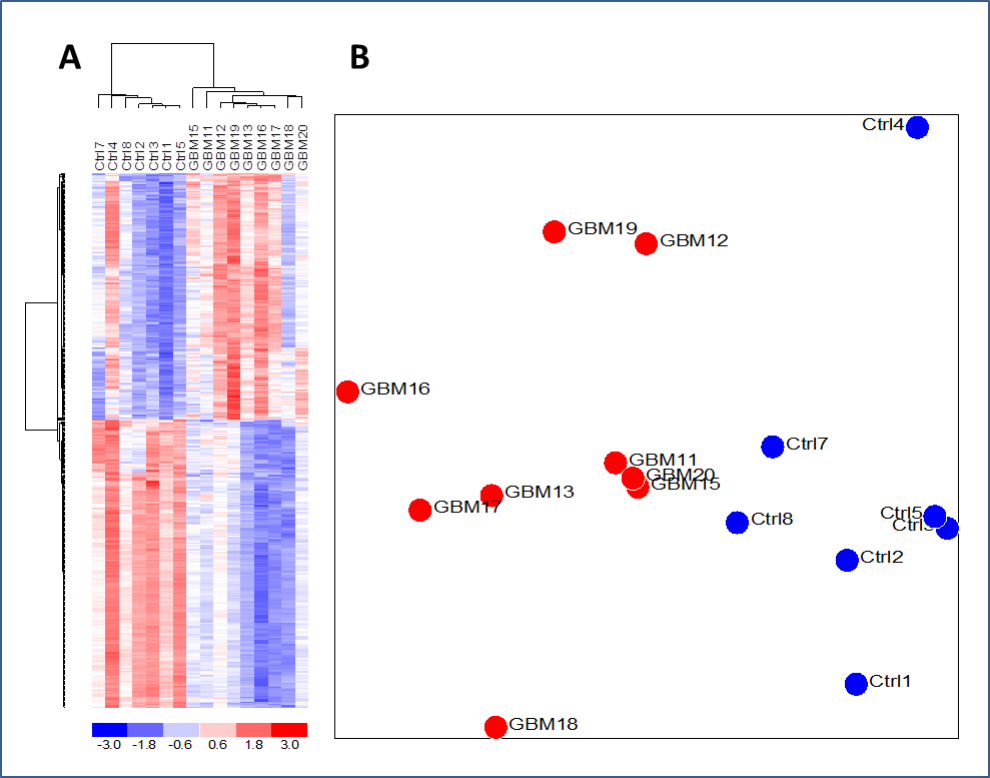
**Analysis of the 400 most dysregulated genes**. The 200 most down- and up-regulated genes, respectively, with p < 0.05 in all three normalizations after correction for False Discovery Rate were used (see Additional file 2: Table S1 and Table S2). A) Cluster analysis, B) PCA plot.

**Figure 3:**
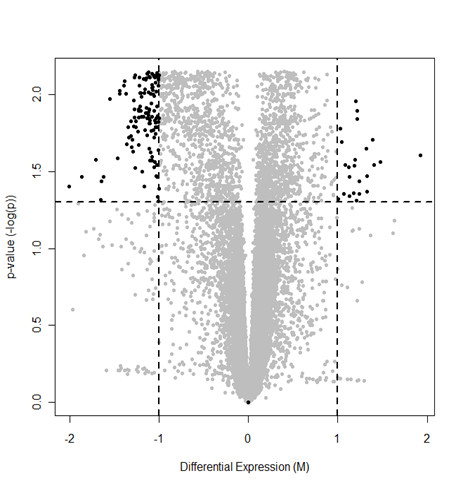
**Volcano plot of the False Discovery Rate (FDR) corrected p-values from a *t*-test between the two sample groups after background subtraction and quartile normalization**. Genes above the horizontal dashed line have *p *< 0.05 after FDR correction. It is evident that substantially more genes are significantly down-regulated (121 genes in upper left corner, > 2-fold) than up-regulated (24 genes in upper right corner, > 2-fold).

With the observation of differences between GBM and control RNA expression patterns, we sought to identify the genes that best separated the two groups by conducting gene-by-gene t-tests between the groups on all genes in the full 44 K data set and corrected the resulting p-values for False Discovery Rate (FDR) by applying the Benjamini and Hochberg algorithm [20]. The 25 most down- and up-regulated genes are listed in Table 1 and more extensive lists of the 200 most down- and up-regulated genes are available in Additional file 2: Table S2 and Table S3, respectively. From the top dendrogram in Figure 1A, which was prepared without the use of p-values, it would appear that the GBM samples separate into two distinct sub-groups. However, when we performed a gene-by-gene *t*-test between these two apparent groups of GBM samples no significant genes were left that met the *p *< 0.05 criteria for statistical significance after application of Benjamini and Hochberg correction for FDR. Similarly, when the genes in Additional file 2: Table S2 and Table S3 were used together for cluster analysis, there was a clear separation of GBMs from controls as seen in Figure 2, but the two distinct groups of GBM samples were no longer apparent. From Figure 3, which shows a volcano plot of the FDR corrected p-values against the level of differential expression for each gene, it is evident that substantially more genes were found to be significantly down-regulated (121 genes) than up-regulated (24 genes) in the GBM samples compared to controls.

**Table 1:** Down-and up-regulated genes passing the criteria described in "Results".

Down-regulated genes			
**No**	**Gene Symbol**	**Fold**	**Description**

**1**	TMSL3	-4.21	Homo sapiens thymosin-like 3 (TMSL3), mRNA [NM_183049]

**2**	A_24_P530977	-4.00	Unknown

**3**	GNG11	-3.86	Homo sapiens guanine nucleotide binding protein (G protein), gamma 11 (GNG11), mRNA [NM_004126]

**4**	RPS4Y2	-3.80	Homo sapiens ribosomal protein S4, Y-linked 2 (RPS4Y2), mRNA [NM_001039567]

**5**	RGS10	-3.63	Homo sapiens regulator of G-protein signalling 10 (RGS10), transcript variant 1, mRNA [NM_001005339]

**6**	RPS4Y1	-3.62	Homo sapiens ribosomal protein S4, Y-linked 1 (RPS4Y1), mRNA [NM_001008]

**7**	B2M	-3.60	Homo sapiens beta-2-microglobulin (B2M), mRNA [NM_004048]

**8**	CCL5	-3.35	Homo sapiens chemokine (C-C motif) ligand 5 (CCL5), mRNA [NM_002985]

**9**	AL049447	-3.35	Homo sapiens mRNA; cDNA DKFZp586A0722 (from clone DKFZp586A0722). [AL049447]

**10**	GPX1	-3.27	Homo sapiens glutathione peroxidase 1 (GPX1), transcript variant 2, mRNA [NM_201397]

**11**	RPS29	-3.12	Homo sapiens ribosomal protein S29 (RPS29), transcript variant 1, mRNA [NM_001032]

**12**	IER2	-3.12	Homo sapiens immediate early response 2 (IER2), mRNA [NM_004907]

**13**	RAP1B	-2.94	Homo sapiens RAP1B, member of RAS oncogene family (RAP1B), transcript variant 1, mRNA [NM_015646]

**14**	MMD	-2.93	Homo sapiens monocyte to macrophage differentiation-associated (MMD), mRNA [NM_012329]

**15**	MAX	-2.89	Homo sapiens MYC associated factor × (MAX), transcript variant 3, mRNA [NM_145113]

**16**	TMEM111	-2.87	Homo sapiens transmembrane protein 111 (TMEM111), mRNA [NM_018447]

**17**	RPS29	-2.82	Homo sapiens ribosomal protein S29 (RPS29), transcript variant 1, mRNA [NM_001032]

**18**	RPL19	-2.81	Homo sapiens ribosomal protein L19 (RPL19), mRNA [NM_000981]

**19**	RPL13	-2.80	Homo sapiens ribosomal protein L13 (RPL13), transcript variant 2, mRNA [NM_033251]

**20**	LOC392497	-2.78	PREDICTED: Homo sapiens similar to 40S ribosomal protein S6 (LOC392497), mRNA [XR_018138]

**21**	MRCL3	-2.74	Homo sapiens myosin regulatory light chain MRCL3 (MRCL3), mRNA [NM_006471]

**22**	RPL30	-2.72	Homo sapiens ribosomal protein L30 (RPL30), mRNA [NM_000989]

**23**	RPS27	-2.70	Homo sapiens ribosomal protein S27 (metallopanstimulin 1) (RPS27), mRNA [NM_001030]

**24**	ENST00000337102	-2.70	40S ribosomal protein S21. [Source:Uniprot/SWISSPROT;Acc:P63220] [ENST00000337102]

**25**	RPA1	-2.68	Homo sapiens replication protein A1, 70 kDa (RPA1), mRNA [NM_002945]

**Up-regulated genes**			

**No**	**Gene Symbol**	**Fold**	**Description**

**1**	CV575560	3.80	oe37f10.y1 Human keratoconus cornea, unamplified, od [CV575560]

**2**	RKHD1	2.32	Homo sapiens ring finger and KH domain containing 1 (RKHD1), mRNA [NM_203304]

**3**	ZNF784	2.65	Homo sapiens zinc finger protein 784 (ZNF784), mRNA [NM_203374]

**4**	SERPINB1	2.62	Leukocyte elastase inhibitor (LEI) (Serpin B1) (Monocyte/neutrophil elastase inhibitor) (M/NEI) (EI).

**5**	LOC390427	2.00	PREDICTED: Homo sapiens similar to TBP-associated factor 15 isoform 1 (LOC390427), mRNA [XM_372498]

**6**	FOXD3	2.32	Homo sapiens forkhead box D3 (FOXD3), mRNA [NM_012183]

**7**	C17orf74	1.97	Homo sapiens chromosome 17 open reading frame 74 (C17orf74), mRNA [NM_175734]

**8**	ENST00000357697	1.67	Unknown

**9**	SLITRK4	2.30	Homo sapiens SLIT and NTRK-like family, member 4 (SLITRK4), mRNA [NM_173078]

**10**	AGRP	2.03	Homo sapiens agouti related protein homolog (mouse) (AGRP), transcript variant 1, mRNA [NM_001138]

**11**	ENST00000359466	1.77	chromosome × open reading frame 18 (CXorf18), misc RNA [RefSeq_dna;Acc:XR_018001] [ENST00000359466]

**12**	THC2760960	1.41	Unknown

**13**	TNXB	2.06	Homo sapiens tenascin XB (TNXB), transcript variant XB, mRNA [NM_019105]

**14**	THC2755576	1.90	ALU1_HUMAN (P39188) Alu subfamily J sequence contamination warning entry, partial (13%) [THC2755576]

**15**	SIN3B	1.60	Homo sapiens SIN3 homolog B, transcription regulator (yeast), complete cds. [BC063531]

**16**	CRLF1	1.66	Homo sapiens cytokine receptor-like factor 1 (CRLF1), mRNA [NM_004750]

**17**	ZNF219	1.97	Homo sapiens zinc finger protein 219 (ZNF219), mRNA [NM_016423]

**18**	THC2654039	1.78	ALU2_HUMAN (P39189) Alu subfamily SB sequence contamination warning entry, partial (4%) [THC2654039]

**19**	AA418814	1.59	AA418814 Soares_NhHMPu_S1 Homo sapiens cDNA clone IMAGE:767978 3', mRNA sequence [AA418814]

**20**	TNRC4	1.72	Homo sapiens trinucleotide repeat containing 4 (TNRC4), mRNA [NM_007185]

**21**	AK098372	1.94	Homo sapiens cDNA FLJ25506 fis, clone CBR05185. [AK098372]

**22**	TNRC6B	1.79	Homo sapiens trinucleotide repeat containing 6B (TNRC6B), transcript variant 1, mRNA [NM_015088]

**23**	HOXA4	1.58	Homo sapiens homeobox A4 (HOXA4), mRNA [NM_002141]

**24**	IL26	1.66	Homo sapiens interleukin 26 (IL26), mRNA [NM_018402]

**25**	THC2718728	1.73	Unknown

"Fold" is calculated as the median value of the fold change in the three described normalization procedures

### Gene ontology analysis

Normalization of array data can be done in a number of different ways, each having its own advantages and disadvantages. To reduce the risk that the normalization procedure introduced unintended biases or artifacts, we normalized the data in three different ways, as described in Methods. For gene ontology analysis we used the top two hundred genes that passed the criteria *p *< 0.05 after FDR-correction in *all *three normalization procedures mentioned above (Additional file 2: Table S2 and Table S3). All genes listed in the tables of this paper met the same selection criteria.

### Down-regulated genes in serum microvesicles from GBM patients

We analyzed the up- and down-regulated genes independently to try to identify brain- or GBM-specific genes up-regulated in the GBM patients due to the microvesicle shedding activity of the tumour, as well as genes down-regulated in normal cells, such as platelets, lymphocytes and endothelial cells [21] that also shed microvesicles into the blood. Gene ontology analysis of the 200 most down-regulated genes (from -4.21 to -1.92-fold, Additional file 2: Table S2) using the online tool, DAVID [22] revealed that an overwhelming majority of the resulting GO terms are related to ribosome functions, e.g. "Ribosome", "Translational Elongation" and "RNA Binding". The most significant cluster had an enrichment score of 54.82 and contained 115 of the 200 input genes, most of which were mRNAs coding for ribosomal proteins in both the large and small subunits of the ribosome (e.g. *RPL11, RPS29, RPLP1, RPS27A*, etc.). These genes have previously been shown to be very highly expressed in lymphocytes relative to other blood cells [23,24]. Evaluation of the percentage of lymphocytes in the white blood cell (WBC) counts in the GBM patients used in this study, showed that 7 out of the 9 patients used for array analysis and 9 out of the 10 patients used for qPCR validation had values of 3-19% lymphocytes, which is well below the reference interval for normal healthy individuals of 22-28% [25] and is consistent with immunosuppression observed in most GBM patients [26]. Unfortunately, the de-identified normal control samples used for this study were obtained through a blood bank so no detailed information is available on their blood cell counts.

### Up-regulated genes in microvesicles from GBM patients

When we left out the down-regulated genes and attempted unsupervised clustering using only genes that were up-regulated in microvesicles from GBM patients (Table 1/Additional file 2: Table S3), no clear separation of the GBMs from the normal controls was evident. Further, gene ontology analysis of the up-regulated genes in Table 1/Additional file 2: Table S3 resulted in clusters and GO terms with enrichment scores substantially lower (< 3) than for the down-regulated genes in Table 1/Additional file 2: Table S2 (> 50).

### Validation analysis by qRT-PCR

We moved on to see if some of the dysregulated genes from the microarray could be validated as dysregulated by qRT-PCR in an independent set of 10 patients and 10 controls. We selected individual up-regulated genes from the lists in Table 1/Additional file 2: Table S3 in search of markers associated with GBM tumours. We sought to identify mRNAs up-regulated in the microvesicles in peripheral blood from GBM patients, which were expressed at low to undetectable levels in blood cells, and elevated in either GBM or normal brain cells. We reasoned that since the majority of microvesicles in blood are believed to arise from platelets [27] and other blood cells, the RNA expression of these cells could serve as an approximation of the normal blood microvesicle RNA profile. We compared public datasets in Gene Expression Omnibus (GEO) for GBM (GSE15824) to those of platelets (GSE11524) and PBMCs (GSE22224) from normal controls to derive a list of "GBM-like" genes highly expressed in GBM, but low or absent in platelets and PBMCs and similarly a list of "blood cell-like" genes highly expressed in platelets and PBMCs. When we compared the "GBM-like" genes to the genes found to be up-regulated in GBM patient blood microvesicle RNA in our own dataset, we found no overlap to support GBM tumour origin of these RNAs. On the other hand, many of the "blood cell-like" genes were the same as those found in our study to be down-regulated in GBM patients relative to normal controls (data not shown).

**Figure 4:**
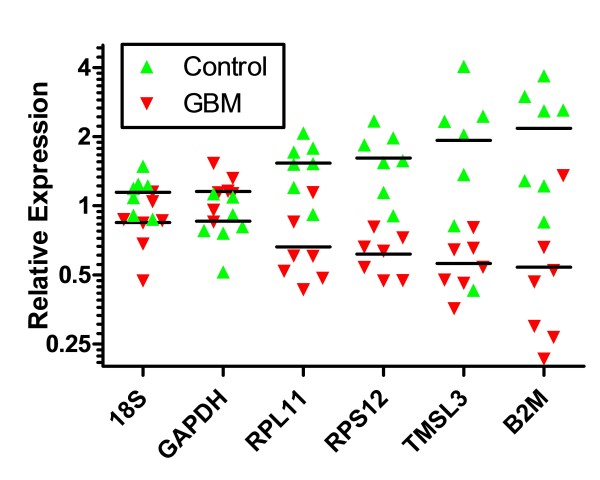
**Validation by qRT-PCR of down-regulated genes**. Gene expression levels were normalized to the combined expression of GAPDH and 18S. Lines represent median values for the GBM and control samples, respectively.

**Figure 5:**
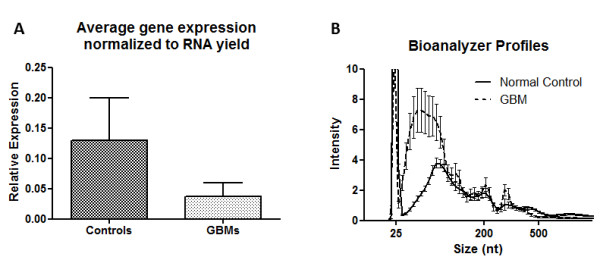
**Serum microvesicle RNA from patients with glioblastoma multiforme has an overabundance of RNA with size <500 nt**. (A) Gene expression relative to the amount of microvesicle RNA present in serum of GBM patients and normal healthy controls. Bars represent the average expression of 16 genes as measured by qRT-PCR, normalized to the amount of exoRNA isolated from 1 mL of serum. The yield from GBM samples is higher than from controls, but this does not result in higher gene expression. (B) From the Bioanalyzer profiles it appears that the extra RNA in GBM serum is predominantly <300 nt. The plot is showing the mean ± SEM (N = 10 GBM and 10 controls). The non-visible parts of the profiles (>500 nt) were very similar for GBM and controls

In selecting genes for further qPCR validation we also considered whether genes were represented in the GO clusters and whether they were genes previously determined to have cancer association. The following genes were chosen for validation studies by qRT-PCR: *RPL11, RPS12, TMSL3 *and *B2M *as expected down-regulated genes and *EGFR, ERBB2, SLITRK4, HOXA4, METT5D1, CV575560, TNXB, ALUY, 7SL *and *THC2718728 *as possible up-regulated genes. For normalization of the qRT-PCR data we chose GAPDH, which appeared to be stable in our exoRNA samples judging from the array data, as well as *18S rRNA*. The qRT-PCR validation was performed on a different set of GBM patients (N = 10) and controls (N = 10) than the array data. We were able to confirm the down-regulation of genes associated with ribosome production *RPL11, RPS12, TMSL3 *and *B2M *observed in the arrays as illustrated in Figure 4, but the expected up-regulated genes did not show any significant increase relative to controls by qRT-PCR (data not shown). RNA was extracted from equal volumes of serum (1 mL) from GBM patients and normal controls. The exoRNA content of the GBM patient serum samples used for qRT-PCR validation in this study, (30.0 +/- 8.3 ng/mL serum; N = 10) was significantly higher (*p *< 0.001) than in the normal control serum samples (8.8 +/- 2.2 ng/mL serum; N = 10). For the microarray analysis, the RNA was linearly amplified and the same amount of RNA was hybridized to all arrays. For the qRT-PCR, the data was normalized to the house-keeping genes GAPDH and 18S. Interestingly, when the qRT-PCR data was normalized to the amount of RNA in the sample all genes initially found to be up-regulated by array analysis appeared to be down-regulated in serum microvesicles from GBM patients, as compared to normal controls (Figure 5A). Inspection of the bioanalyzer profiles of the RNA samples used in this study suggested that the major contribution of the increased RNA amounts seemed to stem from a larger peak of small RNA migrating in the range from 25-300 nt on the bioanalyzer RNA chip (Figure 5B).

## Discussion

Our findings demonstrate the feasibility of genome wide gene expression analysis of RNA extracted from the cell-free microvesicle fraction (exoRNA) of frozen biobanked serum samples, and that clear differences can be observed between the exoRNA expression profiles from GBM patients and normal controls. The two major differences between exoRNA from GBM patients vs. normal controls were found to be: 1) a significantly reduced level of mRNAs encoding ribosomal protein genes; and 2) an overall significant up-regulation of RNA amounts in the serum of GBM patients, which seems to be primarily due to a larger fraction < 300 nt and could not be attributed to any of the mRNAs investigated by qRT-PCR.

ExoRNA from tumour microvesicles in serum will always be diluted into the background of exoRNA coming from normal non-malignant cells. Depending on the purpose of the study, normal exoRNA may also generate useful information. Previous studies have indicated that RNA expression patterns in blood cells can change as a response to tumours [7]. To look specifically at the genes dysregulated in the tumour, one would need to enrich the tumour specific microvesicle fraction before extracting the RNA.

Microvesicles are shed by many different cell types into the blood stream, including circulating WBCs, platelets, endothelial cells, and dendritic cells [17]. Shedding has been shown to be higher from tumour cells in culture compared to normal fibroblasts [28] and tumour-derived microvesicle concentrations in serum increase as a function of increased malignancy [29,30]. However, microvesicles of tumour origin circulating in the blood are mixed with microvesicles from other cell sources and it is likely that tumour microvesicles in many cases constitute a relatively small fraction of the total population.

There is a large contribution of microvesicles from normal cells and platelets in blood. In control plasma, for example, platelet-derived microvesicles have been reported to constitute the majority of all microvesicles [27] and although tumour-derived microvesicles increase in serum as a function of degree of malignancy in ovarian cancer [30] and that the total number of microvesicles in blood increase with disease progression [29], it is not clear what proportion of the increased number of vesicles is coming from the tumour vs. from normal cells as a response to the tumour. Thus, down-regulated genes in GBM exoRNA could either be caused by altered biological processes in normal cells as a response to or consequence of the disease, or by large numbers of tumour microvesicles with low levels of particular RNAs.

A major fraction of the genes we observed to be significantly down-regulated in serum microvesicles from GBM patients in this study code for various ribosomal proteins (e.g. *RPL11, RPS29, RPLP1, RPS27A*, etc.). However, these genes were not found to be among the most abundant transcripts in platelets in a study by [31], which we also confirmed in our analysis of the public dataset from GEO (GSE11524). We speculate that these RNAs would therefore also not be abundant in the platelet-derived microvesicles, which constitute a substantial fraction of the plasma microvesicles [27]. However, these genes have been shown to be very highly expressed in lymphocytes relative to other blood cells [23,24] and were also found to be up-regulated in our analysis of the public dataset for PBMC (GSE22224) relative to GBM. This led us to speculate that the lower abundance of these mRNAs in circulating microvesicles from GBM patients may be a consequence of a reduced level of lymphocyte-derived microvesicles in patients as compared to normal controls, since cancer patients are known to often be immune-compromised [26]. Evaluation of the GBM patients in this study confirmed that their lymphocyte counts were lower than the normal reference level interval, but because we were unable to measure lymphocyte counts in the normal controls, it is impossible to completely correlate transcript down-regulation with lymphocyte depletion in the GBM patients. None of the GBM patients had received chemotherapy prior to diagnosis, but several of them were on steroids and seizure medications at the time of blood draw, which are two potential sources of lymphocytopenia. Evaluation of gene expression differences between lymphocytopenia caused by cancer and by medication would require a much larger study of more carefully chosen subjects.

The observation that many ribosomal protein genes are down-regulated in GBM serum microvesicles may prove to be a valuable contributory marker for GBM and other pathological states. Sharma et al. [5] also observed a reduction in many of the same genes in PBMCs including lymphocytes, from breast cancer patients compared to normal controls. The down-regulation of ribosomal protein genes is therefore unlikely to provide a specific GBM component for a diagnostic classification, but may serve as a more generic pathological indicator and can provide confirmatory support when combined with other exoRNA profiles. Interestingly, many ribosomal proteins have extra-ribosomal functions that go beyond the function of protein biosynthesis. Ribosomal protein genes have been shown to have important regulatory functions also in cancer cells. Some ribosomal proteins are tumour suppressors [32], oncogenes or have regulatory functions in tumour progression, invasion and metastasis [33].

The "ribosome" GO-clusters from down-regulated genes had very high enrichment scores from DAVID (> 50) compared to any of the clusters obtained with up-regulated genes (< 2.5), confirming that the patterns of down-regulated genes were much more significant. For gene ontology analysis it is important to analyze a relatively large number of genes (e.g. 100 [22]) in order to avoid stochastic errors, e.g. if a list of 10 genes is analyzed, the presence of a single gene (10%) of a certain class or belonging to a certain pathway will appear overrepresented compared to the prevalence in the genome, when in fact this is just a sampling artifact. The lack of strong GO associations between the up-regulated genes in our study does not mean that individual genes might not be strongly dysregulated, significant and predictive in GBM exoRNA, but simply that the up-regulated genes do not appear to be associated by already known relationships. Up-regulation of specific genes in exoRNA from GBM patients could be a biological response from normal cells to the presence of the tumour, but they could also be specifically derived from the tumour cells and as such be useful as markers of the tumour. However, identifying uniquely elevated levels of tumour-derived exoRNAs in a high background of normal exoRNA from other serum microvesicle sources is more challenging than identifying systemic changes, such as the depressed lymphocyte count and ribosomal protein RNA levels.

The observation that the up-regulated genes chosen for qRT-PCR validation was not verified may be attributed to the fact that we used two different sets of serum samples for the microarray analysis and the qRT-PCR validation, making borderline significant increases difficult to confirm. Similarly, the positive validation of the down-regulated genes in a separate set of samples makes this observation stronger.

We observed that, coinciding with the significantly increased amounts of microvesicle RNA in GBM patient serum compared to normal controls, the expression levels of all the genes we investigated by qRT-PCR were lower than in normal controls when normalized to total RNA amount (Figure 5). It is likely that this is a general trend across most mRNAs, and since all these genes are down-regulated other transcripts must be up-regulated to account for the overall increase in RNA amounts.

The Bioanalyzer profiles of the different RNA serum samples suggest that this up-regulation of RNA in the GBM patients mainly falls in the range of < 300 nt. This could explain why the majority of the tumour exoRNA transcripts appeared down-regulated on the array. If the < 300 nt RNAs do not contain sequences belonging to the coding genes, they would not have been picked up by the microarray and the mRNA-fraction recognized by the capture probes would be relatively smaller since the same amount of input RNA was used for hybridization. There are many RNA species that are not covered by the microarray, including miRNA (mature and precursors), repetitive elements and other non-coding RNAs, as well as single stranded DNA [28,34].

Balaj et al. [28] showed that human endogenous retroviruses and other transposable elements, as well as single stranded DNA fragments are very abundant in microvesicles from cancer cells, as compared to normal fibroblasts, including Alu, LINE and HERV sequences and it is likely that these and other non-coding RNAs are contributing to the increased amounts of RNA we observe in GBM patient serum (single stranded DNA is also detected on the Bioanalyzer RNA chip). We tested a single transposable element Alu-Y by qRT-PCR and found it to be extremely abundant in the microvesicles, but did not observe any differential expression between the GBMs and normal controls. The human genome consists of about 40% retrotransposon sequences [35], and there is an increasing number of publications showing the dysregulation of transposable elements and other non-coding RNAs in cancer [36-38].

The very existence of the peak of < 500 nt RNAs in GBM serum exoRNA make it a fertile ground for biomarker discovery, and it warrants further investigation to establish the exact nature and distribution of the nucleic acid species contained in this fraction. A number of regulatory non-coding RNAs are transcribed off coding elements [39]. Collectively, down-regulation of specific RNAs and up-regulation of RNA levels in exosomes/microvesicles from serum of GBM patients, as compared to controls provides promising biomarkers. In addition, in other studies, tumour mutant mRNAs [18] and elevated oncogene mRNA [28] have been detected in serum exosomes/microvesicles from human GBM patients and mice bearing medulloblastoma tumours, respectively. Tumour mutant RNA from prostate cancer patients can also be found in urine exosomes/microvesicles [40]. More detailed analysis of exoRNA released by tumour cells into serum should be possible as isolation methods are developed with tumour specific surface markers for different types of cancer, e.g. with magnetic activated sorting [30] and microfluidic capture [41].

## Conclusions

Currently, there are no biomarkers for gliomas to distinguish tumor recurrence from radiation necrosis or to monitor tumor response to therapy. Here we demonstrate that frozen, biobanked glioblastoma serum microvesicles contain RNA in amounts and qualities sufficient to perform qPCR and microarray analysis, and we show that glioma exoRNA profiles are unique compared to tumor free individuals. The most significant expression differences pertained to down-regulated genes in the GBM patient exoRNA, which we were able to validate by qRT-PCR. However, overall yields of exoRNA from GBM patient serum microvesicles was higher than yields from normal controls, but the additional RNA was primarily of size < 500 nt, evident by a peak of small RNAs on the bioanalyzer profiles. Gene ontology analysis of the down-regulated genes indicated these are primarily mRNAs coding for ribosomal proteins and other genes related to ribosome production. The observation may be explained by a reduced contribution of exoRNA from lymphocytes, which have previously been reported to have high expression levels of these transcripts. This theory was supported by the lymphocyte counts in the blood of the patients investigated in this study, which was substantially lower than the normal reference interval.

## Abbreviations

GBM: Glioblastoma multiforme; MGMT: Methyl guanidine methyl transferase; PBMC: Peripheral blood mononuclear cells; MiRNA: MicroRNA; r.t: Room temperature; VSN: Variance stabilized normalization; WBC: White blood cell; GEO: Gene expression omnibus; FDR: False discovery rate; qRT-PCR: Quantitative reverse transcriptase PCR.

## Competing interests

J.S. and M.N. are inventors on the exosome technology used in this study which has been licensed to Exosome Diagnostics, Inc. They hold equity in, and are now employees of that company. Dr. Breakefield is on the Scientific Advisory Board of the company for which she receives cash compensation. None of the other authors have any competing financial interests to declare.

## Authors' contributions

MN performed the data analysis and prepared the manuscript, LB and TL performed the qPCR analysis, AS participated in the array data analysis, BC and FH collected the samples and clinical data, LZ prepared serum from the clinical samples, JS, XOB designed the study and helped draft the manuscript. All authors read and approved the final manuscript.

## Pre-publication history

The pre-publication history for this paper can be accessed here:

http://www.biomedcentral.com/1471-2407/12/22/prepub

## Supplementary Material

Additional file 1 **This file describes the R/BioConductor commands used to analyze the raw data deposited in GEO with accession# GSE24084**. Click here for file

Additional file 2 **Table S1: **Top 200 most down-regulated genes with p < 0.05 in all three normalizations after correction for False Discovery Rate. **Table S2: **Top 200 most up-regulated genes with *p *< 0.05 in all three normalizations after correction for False Discovery Rate. **Table S3: **Sequences and ABI IDs of TaqMan qPCR assays. Click here for file
